# The Whole-Genome Sequence of *Bacillus velezensis* Strain SB1216 Isolated from the Great Salt Plains of Oklahoma Reveals the Presence of a Novel Extracellular RNase with Antitumor Activity

**DOI:** 10.1128/genomeA.01343-17

**Published:** 2017-11-22

**Authors:** Daya Marasini, Carolyn R. Cornell, Opeoluwa Oyewole, Robert J. Sheaff, Mohamed K. Fakhr

**Affiliations:** a Department of Biological Science, The University of Tulsa, Tulsa, Oklahoma, USA; b Department of Chemistry and Biochemistry, The University of Tulsa, Tulsa, Oklahoma, USA

## Abstract

The whole-genome sequence of *Bacillus velezensis* strain SB1216, isolated from the Great Salt Plains of Oklahoma, showed the presence of a 3,814,720-bp circular chromosome and no plasmids. The presence of a novel 870-bp extracellular RNase gene is predicted to be responsible for this strain’s antitumor activity.

## GENOME ANNOUNCEMENT

*Bacillus velezensis* is the later heterotypic synonym of *B. amyloliquefaciens* (1). The complete genome sequences of the plant-associated and antibiotic-producing *B. amyloliquefaciens* strains were known to have circular chromosomes of approximately 4.0 Mb in size (2, 3). The genome of *B. velezensis*, known to have a broad inhibitory spectrum against plant pathogens, contains one circular chromosome of around 4.0 Mb in size (4). An extracellular RNase from *B. amyloliquefaciens* called barnase has been reported to have cytotoxic activity against cancer cells (5, 6).

Here, we announce the whole-genome sequence of *B. velezensis* strain SB1216, which was shown to have antitumor activity. This particular bacterial strain was previously isolated from the Great Salt Plains of Oklahoma (7) and produces a novel ~32-kDa extracellular RNase that was proved to have antitumor activity against human cancer cell lines, including breast cancer cells (8). To our knowledge, this is the first whole-genome sequence to be published for a bacterium isolated from the Great Salt Plains of Oklahoma.

DNA isolation was performed using a modified protocol for Gram-positive bacteria from a 48-h liquid culture of tryptic soya broth using a DNeasy blood and tissue kit (Qiagen, Valencia, CA, USA). The isolated DNA was subjected to next-generation sequencing library preparation using a Nextera XT sample preparation kit (Illumina, Inc., San Diego, CA, USA). The genome sequencing was then performed on the MiSeq desktop sequencer using an Illumina V2 reagent kit of 300 cycles with 100× coverage (Illumina, Inc.). The output FASTQ files of the short Illumina reads were then assembled and analyzed using CLC Genomics Workbench version 7.5.1, and final reference-guided contig arrangement was done using the Microbial Genome Finishing Module version 1.4 (Qiagen).

The genome of *B. velezensis* strain SB1216 contained a 3,814,720-bp circular chromosome. The genome sequence did not show the presence of any plasmids, which was confirmed by pulsed-field gel electrophoresis using S1 nuclease, as previously established in our laboratory, to detect large plasmids (9). The complete genome sequence was submitted to GenBank and annotated using the NCBI Prokaryotic Genome Annotation Pipeline and the Rapid Annotations using Subsystems Technology (RAST) server. The RAST annotation revealed the presence of more genes (3,931) than the NCBI annotation (3,683). According to the NCBI annotation, there was a total of 3,683 genes present, out of which 3,610 coding sequences and 3,538 coding genes were found. There were also 72 RNA genes and 72 pseudogenes present. The pseudogenes had frameshifts and internal stops, were incomplete, or had other problems. The G+C ratio was approximately 46.8%. There were various types of genes present, including those for the biosynthesis of antibiotics, drug resistance, and drug transport. Of special importance for this study, an 870-bp extracellular RNase gene was present and is predicted to code for the ~32-kDa protein providing antitumor activity.

### Accession number(s).

The complete genome sequence of *B. velezensis* strain SB1216 was deposited in GenBank under accession no. CP015417. The version described here is the first version, CP015417.1.
